# Herbal Vitamin C Prevents DNA Oxidation and Modifies the Metabolomic Water Profile of Tilapia (*Oreochromis* spp.)

**DOI:** 10.3390/life12081243

**Published:** 2022-08-16

**Authors:** Moisés Villanueva, Guillermo Espinosa-Reyes, Rogelio Flores-Ramirez, Angel Natanael Rojas-Velazquez, Juan Carlos García López, Anayeli Vazquez-Valladolid, José Alejandro Roque-Jimenez, German D. Mendoza-Martinez, Pedro A. Hernandez-Garcia, Monika Palacios-Martinez, Alfonso J. Chay-Canul, Héctor A. Lee-Rangel

**Affiliations:** 1 Facultad de Agronomía y Veterinaria, Centro de Biociencias, Instituto de Investigaciones en Zonas Desérticas, Universidad Autónoma de San Luis Potosí, S.L.P., Soledad de Graciano Sánchez 78000, Mexico; 2 Facultad de Medicina—CIACYT, Centro de Investigación Aplicada en Ambiente y Salud, Universidad Autónoma de San Luis Potosí, Lomas Segunda Sección, San Luis Potosí 78210, Mexico; 3 Departamento de Producción Animal, Universidad Autónoma Metropolitana—Xochimilco, CDMX, Mexico City 04960, Mexico; 4 Centro Universitario UAEM Amecameca, Universidad Autónoma del Estado de México, Carretera Federal Amecameca-Ayapango km 2.5, Amecameca de Juárez 56900, Mexico; 5 División Académica de Ciencias Agropecuarias, Universidad Juárez Autónoma de Tabasco, Carretera Villahermosa-Teapa, km 25, R/A. La Huasteca 2ª Sección, Villahermosa 86280, Mexico

**Keywords:** herbal vitamin C, DNA damage, tilapia

## Abstract

This experiment aimed to evaluate the effects of herbal vitamin C at different levels on tilapia (*Oreochromis* spp.) growth, potential DNA damage, and the metabolomic profile of water effluent. Forty-five tilapias were housed in separate plastic tanks (80 L), and these were randomly assigned to three treatments: (a) a commercial diet (CD) only; (Nutripec Purina^®^); (b) the commercial diet plus 250 mg of herbal vitamin C (HVC)/kg (CD250); and (c) the commercial diet plus 500 mg of HVC/kg (CD500). Biometric measurements were taken each week, blood samples were collected from the caudal vein on the final day, and water effluent was taken each week and immediately frozen (−80 °C) until further analysis (gas chromatography/mass spectrometry (GC/MS) systems). Data were completely randomized with a 2 × 2 factorial arrangement of treatments. Upon including herbal vitamin C, the final BW (*p* = 0.05) and BWG (*p* = 0.06) increased linearly. Herbal vitamin C decreases DNA damage (*p* ≥ 0.05). PLS-DA showed a 41.6% variation between treatments in the water samples. Fifteen metabolites had the best association between treatments, with a stronger correlation with CD500. Herbal vitamin C could improve fish performance, prevent DNA damage, and influence changes in the metabolomic profile of the water.

## 1. Introduction

In 2050, the population will reach over ten billion people [1]; however, the land, water, and food ingredients to support this growth are limited. Scientists and world leaders are working together to make strategic decisions, to establish policies to utilize the available resources and maximize production for food sovereignty [2]. Aquaculture is a potential food system in different countries [3]. Various researchers are convinced that fish is one of the cheapest and most promising sources of food production. Additionally, in human health, it has been identified that people could easily digest 93.2% and 93.7% of fish protein and fat, respectively [3]. The Food and Agriculture Organization reports that the demand for intensifying the productivity of available finfish species has become urgent [4]. For this reason, different fish species have been established on intensive systems. Nevertheless, the viability of fish production has also been evaluated according to consumer demand, food quality, prices, cost of production, resistance to farming stressors, and profitability [5].

Increasing intensive aquaculture means multiplex changes in farming conditions that may affect the immunity of the fish, such as environmental conditions, husbandry, genetics, nutrition, and exposure to bacterial fish pathogens. Thus, many fish farming conditions are stress-related, predisposing the fish to diverse diseases [6]. Consequently, commercial fish health management strategies to meet the disease challenges often include feed-supplemented immune stimulants and stress-alleviating nutrients and additives [7]. To increase commercial aquaculture, diagnosing and treating infectious diseases are crucial to reducing illness caused by bacteria, viruses, or parasites, as illness causes economic loss and threatens the future of aquaculture [8,9]. Disinfectants, antibiotics, and chemotherapeutics are commonly used against fish diseases [10]. However, negative consequences have led to the development of antibiotic-resistant bacteria, residual antibiotics in fish products, genotoxicity and cytotoxicity by chemicals, and general environmental pollution [11,12,13,14].

Nevertheless, Regulation 1831/2003/EC, established by the European Union in 2006, imposes a ban on sub-therapeutic antibiotics as growth promoters; therefore, alternatives to traditional antibiotics in aquaculture practices have consequently received much attention in recent years [15]. These tendencies also correlate with the growing sensitivity of consumers, who increasingly demand eco-friendly farming practices and chemically uncontaminated food products [16].

The use of plants with medicinal properties in aquaculture has been practiced for a long time in Asia, and as traditional medicine in China, Sri Lanka, India, Pakistan, and Thailand. In other countries, such as Thailand, China, Mexico, and Japan, various plant species have been studied in aquaculture [17]. Thus, alternative sources of feedstuff should be evaluated to maximize animal production [18,19]. Recently, research that focused on using herbal feed additives has reported beneficial effects on livestock [20]. Herbal feed additives have been reported with different bioactive compounds, such as alkaloids, polyphenols, isothiocyanates, tannins, saponins, and terpenoids, all of which can stimulate the immune system [21].

In aquaculture, using herbal immuno-stimulants to strengthen the health status of fish and improve their resistance against challenging pathogens has led to promising results [22]. Polyphenols as secondary plant metabolites are characterized by their potential antioxidant and immune-stimulating functions [23]. Moreover, they can be regarded as natural antibiotics, with a possible application as dietary additives in aquaculture [24]. In addition, it is well known that polyphenol-rich extracts can improve immunity and increase disease resistance in fish [25].

Apparently, some products (such as Power-C^®^) based in a polyherbal mixture contain natural, stable, and highly bioavailable vitamin C. Among non-enzymatic antioxidants, vitamin C is a crucial micronutrient for reducing oxidative stress [26]. Most fish cannot synthesize vitamin C alone, but they can achieve it from exogenous sources. Vitamin C is a powerful antioxidant as it can be oxidized by most free radicals in an aqueous solution and converted into less reactive substances. Therefore, this study aimed to evaluate the effect of herbal vitamin C supplementation in a closed aquaculture model to determine cellular damage from oxidative stress on tilapia.

## 2. Materials and Methods

### 2.1. Herbal Vitamin C Characterization

The herbal vitamin C used (HVC; C-Power^®^, Nuproxa, LTD, Etoy, Switzerland) is a commercial herbal formula. The manufacturer states that: “*In Herbal C, there is natural esterification of an active ingredient moiety at 2,3 enediol structure. Moreover, dual esterification of active moieties gives better stability to Herbal C as compared to synthetic esterified Vitamin C. Further, the greater number of hydroxyl groups containing many incipient H atoms in the active moiety of Herbal C provides better solubility and hence better bioavailability as well as higher activity to Herbal C*”. The recommended dosage is 100 g of herbal vitamin C, to replace 100 g of synthetic vitamin C. The extraction of organic compounds was performed using an ultrasonic processor (GEX130, 115 V 50/60 Hz) equipped with a 3 mm titanium tip and mechanical stirrers (Cole-Parmer, Vernon Hills, IL, USA). Briefly, 1 gram of the HVC was mixed using 10 mL of acetone. Following this, the organic phase was separated, concentrated to 1 mL of extracted mixture, and evaporated (Zymark, Turbovap LV Concentration Evapotarot, Hopkinton, MA, USA) for the last step of the analysis. The characterization of the organic compounds was performed with gas chromatography (GC-HP 6890) coupled with mass spectrophotometry (MSHP 5973). The mass spectrophotometry was programmed in SCAN mode (50–500 *m*/*z*) to identify compounds.

### 2.2. Experimental Site and Animals

The research was conducted at the Centro de Biociencias of UASLP, Mexico. The protocol was submitted to the Postgraduate Committee to evaluate the procedures; it was reviewed and approved because it is in accordance with the regulations of the Mexican government.

Forty-five tilapias (*Oreochromis* spp.), sexually reverted with a mean body weight (BW) of 175.6 ± 16.6 g, were randomly assigned to 1 of 3 growing treatments, housed in separate plastic tanks (80 L) containing aerated recirculated freshwater, and reared at 25 °C with a 12:12 h light:dark cycle maintained by an automatic timer. Water pH was maintained between 7.0 and 7.5, and dissolved oxygen was maintained between 7 and 8 mg/L. The tilapias were fed with a commercial diet (Nutripec Purina^®^), which contains 32% protein and 6% fat.

Herbal vitamin C (C-power Nuproxa^®^) was added to the feed by an additive, 3 dosages were used as treatments: (a) commercial diet (CD) only (Nutripec Purina^®^); (b) commercial diet plus 250 mg of HVC/kg (CD250); and (c) commercial diet plus 500 mg of HVC/kg (CD500). At 0800, 1500, and 2100, the fish were fed their respective diets by apparent satiation for 35 days. At the beginning of the experiment and each week thereafter, the fish were weighed and measured individually.

### 2.3. Comet Assay

After completing the experiments, blood samples were collected from the caudal vein of the fish. A single-cell gel electrophoresis was then performed as described by Singh et al. [27] with slight modifications by González-Mille et al. [28]. A total of 30 µL of blood was mixed with 225 µL of agarose; this was added onto the slides of low melting point to a previously preprepared coat of agarose (Sigma-Aldrich, St. Louis, MO, USA) at 0.5%. The agarose was solidified and sat in lysis solution (10 mM TrisHCl, 2.5 M NaCl, and 0.1 M Na2EDTA (pH 10)), with 10% DMSO and 1% Triton X-100.

**Figure 1:**
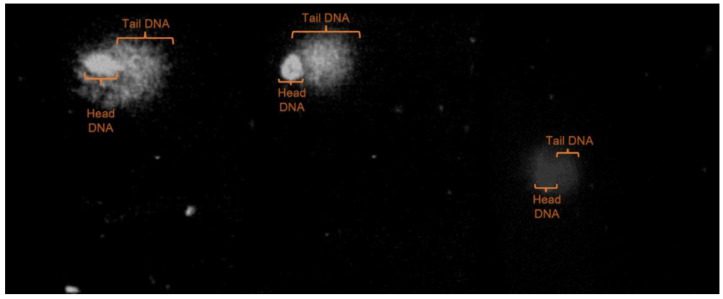
Microscope image observed single-cell electrophoresis (comet assay) of the erythrocytes from tilapia (*Oreochromis niloticus*) whole blood with different levels of DNA damage.

The lysis lasted 24 h at 4 °C. The incubation of slides was performed in an alkaline buffer for 5 min. Electrophoresis was completed in the same buffer (pH > 13) for 5 min at 25 V and 300 mA. Low light and a temperature of 4 °C were the conditions for performing the procedures; the slides were rinsed with Tris-HCl buffer (pH 7.5), and ethanol was used to dehydrate them. The staining was carried out by ethidium bromide (Sigma-Aldrich, St. Louis, MO, USA) (0.05 mM). One hundred cells (duplication of 50 randomly selected cell nuclei) were analyzed to identify the level of DNA damage using an epifluorescent microscope (Nikon Eclipse E400, Tokyo, Japan) (Figure 1). The Olive tail moment and tail length were measured through image analysis (Kom-et, version 4; Kinetic Imaging Ltd., Bromborough, UK).

### 2.4. Volatile Compounds of Water Effluent

A volume of 5 mL of water effluent was placed in a 20 mL vial sealed with a silicone cap (Agilent^®^ 75.5 × 22.5, Santa Clara, CA, USA). A 2 cm, 100 μm film thickness polydimethylsiloxane (PDMS Stableflex SPME fiber (Supelco)) was used for the sample preprocessing; the SPME fiber was activated at 270 °C following the manufacturer’s instructions. The PDMS fiber was exposed to the sample with continuous stirring at 60 °C for 15 min. Following this, the fiber was analyzed using a gas chromatograph, Agilent 6890^®^, coupled to a mass spectrometry detector, Agilent 5975^®^, in electron impact ionization mode. The injection port was operated in the split less mode with a 0.75 mm liner made of glass wool. The injection port temperature was 220 °C; helium was used as carrier gas at a pressure of 36 psi with a constant flow of 0.9 mL min^−1^.

Chromatographic separation was performed using an HP 5MS (60 m × 0.25 mm × 0.25 μm) column (Agilent). The setting of the oven was as follows: 70 °C (initial; 0 min^−1^), 180 °C (10 min^−1^), and 200 °C (5 min^−1^), for a run time of 15 min. The tune parameters were: 35 μA; energy: 69.9. The SCAN mode (50–500 *m*/*z*) was employed for the identification of the compounds. The peak areas were taken to be the relative abundances of each volatile compound. The compounds were identified by the NIST 14 library. Finally, the results were obtained and processed using Chemstation Software (Agilent).

### 2.5. Data Analysis

A complete randomized design was used to analyze the SAS Proc Mixed (9.4) sentence [22]. The treatment was considered a fixed effect, and the fish were the random effect. A covariate (initial BW) was included in the productive data. Orthogonal polynomial contrasts were used to verify linear or quadratic effects for HVC inclusion. The data are presented as LSmean and SEM.

The multivariate analysis of metabolites was processed using MetaboAnalyst 5.0. The metabolite data were transformed using the generalized log transformation, and then range-scaled to correct for heteroskedasticity and to reduce mask effects. A partial least squares discriminant analysis (PLS-DA) and a variable importance in projection (VIP) were performed using R to identify the differential metabolites among groups, and to rank them according to their importance in discriminating groups.

## 3. Results

**Figure 2:**
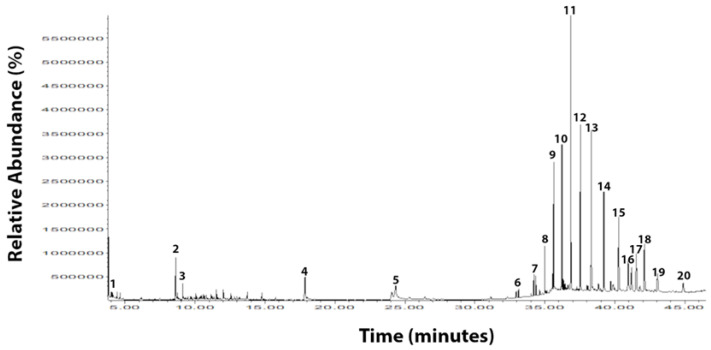
Volatile components in herbal vitamin C analyzed by CG-MS: 1. Toluene; 2. Thymol; 3. Phenol, 2-methoxy-3-(2-propenyl); 4. N-Hexadecanoic acid; 5. 9-Octadecenoic acid; 6. Tetracosane; 7. Nonadecane; 8. Hexacosane; 9. Heptacosane; 10. Docosane; 11. Eicosane; 12. Triacontane; 13. Nonacosane; 14. Nonadecane; 15. Heneicosane, 11-decyl; 16. Gamma-sitosterol; 17. 4,4,6a,6b,8a,11,11,14b-Octamethyl-1,4,4a,5,6,6a,6b,7,8,8a,9,10,11,12,12a,14,14a,14b-octadecahydro-2H-picen-3-one; 18. Taraxasterol; 19. Hexatriacontane; 20. Eicosane.

Seventy-one compounds were detected in HVC, but according to the Kovat’s index database, 20 major relevance compounds were identified in HVC (Figure 2), including aromas, alcohols, aldehydes, and phenolics, some with nutraceutical properties.

Table 1 shows the growth performance of the fish supplemented with HVC. The best performance was observed for the animals that received the HVC compared to the commercial diet. The inclusion of HVC linearly improved (*p* = 0.05) the final BW; therefore, the BWG increased linearly (*p* = 0.06).

The DNA damage determined by the comet technique showed effects (*p* ≤ 0.05; Table 2) over time through decreases in the tail DNA, Olive tail moment, and tail length on each passing week. It was observed that there was minor DNA damage in fish blood regardless of whether they were supplemented or not. During Weeks one and two, an effect (*p* ≤ 0.05; Table 2) of supplementation was observed concerning the control for all variables; in Weeks three and four, significant differences (*p* ≤ 0.05; Table 2) were observed between treatments as well as in all variables for the dose, presenting minor damage when supplementing with a dose of 500 mg/kg DM.

**Figure 3:**
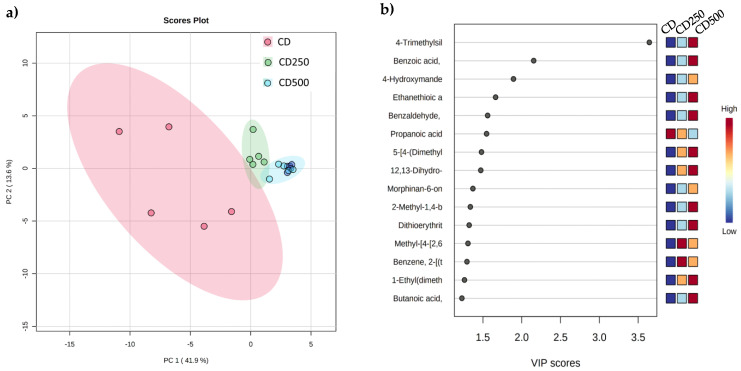
(**a**) Partial least squares discriminant analysis (PLS-DA) score plot in 2D graphs using the concentration of all metabolites quantified by groups; (**b**) variable importance in projection (VIP) plot analyses obtained from the water effluent.

The metabolomic profiles of the water effluents (Figure 3a) showed that the treatments are different (Figure 3). The comparison was performed using a PLS-DA to elucidate the specific differences between groups. The samples were classified within their specific treatments and were analyzed in the MetaboAnalyst program. The maximum separation between treatments was up to 41.6% variation in the data set in the water samples.

The variable importance for the projection (VIP) plot showed discriminating variables when the values were more significant than one in partial least squares. Figure 3b shows that the metabolites correlated with the treatments. The VIP score shows the 15 metabolites with the best association between treatments. A higher VIP value is associated with a CS. The most correlated metabolites were: 4-trimethylsilyl; benzoic acid; 4-hydroxymande; ethanethioic acid; benzaldehyde; propanoic acid; 5-(4-(dimethyl); 12,13-dihydro; morphinian-6-one; 2-methyl-1,4-b, dithioerythritol; methyl-(4(2,6); benzene,2-((t); 1-ethyl(dimethyl); and butanoic acid.

## 4. Discussion

Functional feed is an innovative concept in the aquaculture industry. It helps to design feed systems that extend beyond satisfying the basic nutritional requirements of the aquaculture organism. Research in this area is worthy of pursuing further and may be very helpful for the industry [29].

The application utilizes natural extracts, such as essential oils, herbal products, aromatic plants, and oleoresins to replace antibiotic growth promoters in terrestrial animal feeds [15]. This application has demonstrated positive effects in feeds for various aquaculture systems [30]. Phytogenic substances in fish diets have the potential to control disease, immune response, and resistance [31], as well as storage quality improvement and the antioxidant properties of fish fillet. They are viable alternatives to antibiotic growth promoters [15].

Our results showed that CD treatment resulted in a lower final weight gain among tilapias than those fed with the HVC diets. Rathore et al. [32] mentioned that dietary vitamin C could improve the growth performance of tilapia fingerlings; the differences in final body weight (FWG) could be due to development stage, cultivation environment, variation in experimental conditions (including levels of nutrient interaction in the treatment diets), and other feed contents, such as other vitamins [33]. However, our experiment was established under controlled conditions (environment, age, weight, gender, and diet). Vitamin C increases intestinal villi development and the number of goblet cells, which could increase the absorptive function of the intestine by supplying a larger surface area for the absorption of nutrients [34], resulting in a significant FBW.

The HVC (C-Power^®^) we used was a dry polyherbal mix based on *Emblica officinalis* and *Ocimum sanctum*; the possible effects on tilapia performance may be due to the secondary metabolites of these herbs. Medicinal herbal or polyherbal extracts, mixes, or powders have been used to promote the growth and survival rates of tilapia species [35]. The results of medicinal herbal extracts on performance in fish species is explained by their wide range of immuno-nutritional constituents, including complex sugars such as polysaccharides. Polysaccharides have prebiotic properties [36] that could increase nutrient digestibility, absorption, and assimilation capacity of an animal through an improved gastrointestinal morphology or digestive system [37]. Thymol is a constituent of HVC; Alagawany et al. [38] mentioned that thyme enhances the absorption of the nutrient in the gut, and thus can lower the prevalence of mortalities and disease via antibacterial actions in the gastrointestinal tract of fish.

In fish feed, some oxidized components can be produced in several feed ingredients, which may affect the oxidative stress and health status of fish [39]. DNA oxidation is the most frequently occurring damage in somatic cells, which is caused by free radicals (FRs) and other reactive DNA species. The cell damage is caused by FRs and other reactive oxygen species (ROS), such as hydrogen peroxide (H_2_O_2_), superoxide (O_2_), hydroxyl radicals (OH), and nitric oxide (NO), among others [40]. Oxidative stress results from the cell’s metabolic processes, such as respiration, biosynthesis and respiration, biodegradation of molecules, and the xenogeneic biotransformation of xenobiotic phagocytic activity [41].

It is important to mention that aquatic animals should have stability between the ROS and antioxidant defenses [42]; some stressor factors such as pollution, osmotic stress, temperature fluctuations, and variability in oxygen concentrations directly affect the free radical chemistry [43]. Organisms develop some strategies to either prevent or repair the effects of oxidative stress and to take care of the ROS, and this prevention comes in the way of enzymes, antioxidants, or non-enzymatic molecules (e.g., ascorbic acid (vitamin C)) [42]. In this sense, vitamin C can intercept reactive electrophilic metabolites due to its marked nucleophilic properties, thus preventing attacks on nucleophilic sites in DNA. Vitamin C is a well-known antioxidant that might inhibit oxidative metabolism; it can also scavenge harmful free radical metabolites or reactive oxygen species [44]. Vitamin C can reduce unstable species of oxygen, nitrogen, and sulfur radicals, in addition to regenerating other antioxidants in the body, such as alpha tocopherol (vitamin E) [45].

Due to their derivatives or extracts, some herbs have been studied to evaluate their effective components such as resveratrol, carvacrol, curcumin, and thymol [46]. In some animal diets they have improved antioxidant indices [38].

Our research showed that some metabolites show greater correlation depending on the treatments. The most correlated metabolites were 4-trimethylsilyl; benzoic acid; 4-hydroxymande; ethanethioic acid; benzaldehyde; propanoic acid; 5-(4-(dimethyl); 12,13-dihydro; morphine-6-one; 2-methyl-1,4-b; dithioerythritol; methyl-(4(2,6); benzene,2-((t); 1-ethyl(dimethyl); and butanoic acid. The CD500 treatment increased the VIP score for the 4-trimethylsilyl metabolite in water effluent. Currently, the functions of the 4-trimethylsilyl metabolite in aquaculture are still unknown. Sardela et al. [47] discussed how the water effluent from the tank may provide valuable information about the metabolites as a model of fish metabolism. According to the National Center for Biotechnology Information [48], the 4-trimethylsilyl metabolite has been shown to be attributable to antimicrobial and anti-inflammatory activities [49]. However, this research could not demonstrate that the origin of this metabolite may be related to fish metabolism by supplementation with vitamin C. Nevertheless, the data from this study showed minor DNA damage in the tilapia when they received a greater amount of the herbal mix as a source of vitamin C. Thus, the current research provides new evidence for a probable metabolite with an anti-inflammatory role. Further investigation is warranted to describe the participation of this metabolite in aquaculture and its origin in the water tank. The CD500 treatment also showed a higher VIP score for benzoic acid.

## 5. Conclusions

The addition of herbal vitamin C to the diets of tilapia improved the final body weight and increased the average daily gain at 35 days. Increasing the dosage prevents DNA oxidation and causes changes in metabolic compounds in the water effluent. HVC prevents DNA damage and leads to changes in metabolomic analysis. HVC could be a viable product to replace commercial vitamin C in aquaculture, although further investigation is required.

## Figures and Tables

**Table 1:** Performance of tilapias supplemented with herbal vitamin C over 35 days.

Item	CD	CD250	CD500	SEM	l	q
Initial BW, g	162	181.75	187.75	9.25	0.12	0.71
Final BW, g	188	215.5	217	7.98	0.05	0.85
BWG, g	26	33.75	29.25	1.31	0.06	0.78
Initial longitude, cm	20.15	21.15	20.95	2.7	0.25	0.26
Final longitude, cm	22.75	23.55	23.47	1.99	0.39	0.68
Difference, cm	2.62	2.45	2.53	1.83	0.16	0.13

CD, commercial diet; CD250, commercial diet + 250 mg HVC/kg; CD500, commercial diet + 500 mg HVC/kg; BW, body weight; BWG, body weight gain; l, lineal effect; q, quadratic effect; SEM, standard error of the mean.

**Table 2:** DNA damage in blood of tilapia supplemented with herbal vitamin C over 35 days.

Item	Period					*p*-Value		
	Week 1	Week 2	Week 3	Week 4	SEM	Time	l	q
**Tail DNA**								
CD	49.5 ^b^	48.68 ^b^	30.45 ^c^	14.14 ^c^	1.76	0.04	0.69	0.86
CD250	3.83 ^a^	2.8 ^a^	2.57 ^b^	5.24 ^b^				
CD500	3.27 ^a^	2.6 ^a^	1.45 ^a^	2.47 ^a^				
**Olive Tail Moment**								
CD	10.98 ^b^	11.89 ^b^	6.93 ^c^	2.94 ^c^	0.48	0.03	0.9	0.93
CD250	0.54 ^a^	0.54 ^a^	0.43 ^b^	0.9 ^b^				
CD500	0.56 ^a^	0.46 ^a^	0.27 ^a^	0.48 ^a^				
**Tail Length**								
CD	24.11 ^b^	29.99 ^b^	16.7 ^c^	9.07 ^c^	0.91	0.05	0.96	0.72
CD250	2.2 ^a^	2.6 ^a^	2.13 ^b^	4.46 ^b^				
CD500	2.82 ^a^	2.58 ^a^	1.52 ^a^	2.36 ^a^				

CD, commercial diet; CD250, commercial diet + 250 mg HVC/kg; CD500, commercial diet + 500 mg HVC/kg; BW, body weight; BWG, body weight gain; l, lineal effect; q, quadratic effect; ^a,b,c^ Values with different letters in a row are different; SEM, standard error of the mean.
